# Correlation between dizziness and impaired glucose metabolism

**DOI:** 10.1016/S1808-8694(15)30970-8

**Published:** 2015-10-19

**Authors:** Adriano Santana Fonseca, Silvia Angeleri Valente Davidsohn

**Affiliations:** a Otorhinolaryngologist-Head and Neck Surgeon - HC/UNICAMP. Assistant Professor of Head and Neck Surgery - Santa Casa de Salvador - Hospital Santa Izabel. Head of the Otolaryngology Department - Hospital Naval de Salvador. Specialist in dysphagia - NOEV/Hospital da Bahia; b Endocrinologist and Metabologist - HC/UNICAMP - CESA/Hospital da Bahia, Head of the Endocrinology Department - Hospital Naval de Salvador, Head of the Endocrinology Clinic - Instituto do Pé Diabético, Salvador, Bahia. Núcleo de Otorrinolaringologia e Estudos da Voz, NOEV, Salvador, Bahia

**Keywords:** diabetes mellitus, sensorioneural hearing loss, vectoelectronystagmography, vertigo, tinnitus

## Abstract

**Introduction:**

Impaired glucose metabolism is characterized by conditions of hypo and hyperglycemia.

**Aim:**

The objective of the present study was to asses whether or not there is a relationship between impaired glucose metabolism and dizziness. In the clinical laboratory settings, patients were examined using vectoelectronystagmography in association with glycemic levels.

**Methods:**

33 patients were divided in 3 groups: diabetics; patients with dizziness and a control group. Results:65% of the patients with dizziness showed impaired glucose metabolism. 40% of the patients with dizziness had alterations in their vectoelectronystagmography results.

**Conclusion:**

Dizziness is a good indicator of glucose metabolism alterations and these may be a good indicator of alterations in vectoelectronystagmography responses. The study of glycemic levels after glucose overexposure is a good prognosis factor to evaluate dizziness and shows the same results as insulin level studies after glucose overexposure.

## INTRODUCTION

Glucose metabolism alterations are characterized by states of hypo and hyperglycemia. There are different diagnostic possibilities to be considered in these cases, for instance: reactive hypoglycemia (secondary to hyperinsulinemia, after glucose overload), glucose intolerance, altered fasting glycemia and diabetes (types 1 and 2)^1^.

All the subgroups of glucose metabolism alteration show damage to different organs^1^.

Besides being the major energy source for homeostasis, glucose also can diffuse easily to different tissues and bears an osmotic potential able to alter the workings of our metabolism^1^.

The cochleo-vestibular organ is part or our body balance control system, the vestibular system. By balance we understand the counterbalanced state of opposite forces that crop up with body movements^2^. The vestibular system integrates the information sent by the peripheral receptors - the eyes, the propioceptors and the labyrinths. Such information is directed towards the vestibular nuclei and from there it is sent to and processed by the central nervous system^3^. The proper functioning of this system depends on the perfect integration of all the information that come from peripheral receptors.

Glucose metabolism supplies the energy necessary to maintain from the endolymphatic and perilymphatic potential difference, all the way to the neuronal transmembrane potential, which will allow this peripheral information to reach the CNS and be properly processed. A glycemia reduction beyond physiological limits reduces the energy supply necessary for a proper functioning of the Na/K pump, which is responsible for the maintenance of transmembrane potentials^1^, ^4^.

A glycemia increase beyond physiological levels generates glucose build up within bodily fluids and its large osmotic potential alters the functioning of all the systems, the vestibular among them^2^, ^4^.

The goal of this paper is to check and see if there is any relationship between glucose metabolism alterations, throughout fasting glicemia and glucose tolerance tests, and diziness.

## MATERIALS AND METHODS

Our initial study included patients who sought the otolaryngologist because of diziness without previous history of diabetes mellitus (group 1) and patients who sought the endocrinologist in order to control Diabetes mellitus, without spontaneous complaints of diziness (group 2).

The control group was formed by patients who sought the otorhinolaryngologist because of nasal issues, without complaints of diziness.

All patients were submitted to a basic questionnaire and had to fill out the Cohen and Kimball Daily Activities Scale and Vestibular Disorders, besides signing an informed consent to participate in this research.

Patients who sought the otorhinolaryngologist, but knew they had glucose metabolism alteration, labyrinth diseases, and cochlear diseases or brought incomplete test results, as well as diabetic patients with previously diagnosed cochlear and labyrinth diseases were excluded from the study.

The study was then carried out in a group of 33 patients, initially divided in 3 subgroups:1) Group of patients who sought the otorhinolaryngologist: 11 patients from 26 to 56 years; mean of 44.7 years - 9 women and 2 men.

2) Group of patients who sought the endocrinologist: 13 patients, between 38 and 75 years, mean age of 59.69 years - 11 women and 2 men.

3) Control group: 9 patients, between 35 and 80 years, mean age of 55 years - 6 women and 3 men.

The groups were clinically assessed and underwent complete audiometric tests, impedance tests, brainstem evoked response audiometry and vectoelectronystagmography. For diabetic patients we also tested their fasting glucose levels, and for the others the glucose tolerance test in 5 different intervals according to O'sullivan's biochemical criteria. Being older and allowing a more accurate follow up, O'sullivan criteria of reactional hypoglycemia were factors used by the gap left by the cutting points disagreement, presented by the most relevant world entities in Diabetes: World Health Organization, American Diabetes Society, International Diabetes Foundation and European Diabetes Association.

In developing this work, we decided to statistically group the patients who complained of diziness, those of glucose metabolism disorders and those with vectoelectronystagmography alterations as distinct subgroups, which would be compared among themselves and among those who did not complain.

## RESULTS

The three groups were made up of 20 patients with either spontaneous or prompted complaint of diziness. Of these, 13 (65%) had glucose metabolism alteration. Now, among the 13 patients of the 3 groups of patients without diziness complaint, 4 (30%) had glucose metabolism alterations. Looking at the results of the vestibular tests, we could see that 40% of the patients who complained of diziness had alterations in their clinical vestibular exam and at vectoelectronystagmography, while 7.5% of the asymptomatic patients had the aforementioned vestibular alterations.

When we individually evaluated the 17 patients with glucose metabolism alterations, we found 6 patients with alterations in their vestibular tests, 32%, compared to 18% of those who had vestibular test alterations and normal glucose metabolism.

## DISCUSSION

The papers that discuss cochleo-vestibular function of patients with glucose metabolism impairment, most of the times, associate hearing loss with the progression of diabetes mellitus. The few papers that assess the balance of patients with glucose metabolism disorder find electronystagmography alterations in 27.1% to 43.8% of patients with glucose metabolism impairment^4^, ^5^, ^6^, ^7^, ^8^, ^9^, ^10^, ^11^, ^12^. These data confirm our findings so far raised in this pilot study. However, most of these papers associate balance disorders with impairments in insulin production and release, calibrated by insulin curve test checked by the insulinemia curve test^4^, ^5^, ^6^, ^7^, ^8^.

The high costs of the insulinemia curve test and the fact that the American Association of Diabetes has not yet standardized it, have led us to carry out its indirect evaluation through the glycemia curve, of which results have so far been similar^6^, ^7^, ^8^, ^9^, ^10^, ^11^, ^12^.

The research that challenge glicemia levels to assess glucose metabolism are based on a large number of patients that have vestibular disorders, such as Ménière syndrome, with curves that suggest hyperinsulinemia and normal glicemia levels. These authors forget, though, that insulin levels for intermediate time points (30, 60, 90 minutes) are being challenged by the main entities that deal with diabetes in the world today. Therefore, now that all standards are being challenged, all studies are valuable, specially those with a control group.

The general populations between 18 and 75 years of age have diziness prevalence percentages of 7-15%. And the populations above 75 years of age have prevalence levels varying between 30 and 35%^1^, ^6^, ^7^, ^8^, ^9^, ^10^, ^11^, ^12^.

The initial group distribution and its evaluation individually and as a whole was very relevant, for it made it clear that a large number of patients who seek the endocrinologist to control their glicemia levels complain of imbalance; in this paper we mention 13 patients with diziness among the 17 patients with glucose metabolism impairment (76%). The life quality of these patients may be enhanced by a multidisciplinary approach. We have also observed that the initial diagnosis of Diabetes Mellitus and other glucose metabolism disorders, which have a potentially lethal risk for patients, may be carried out by the otorhinolaryngologist - if he/she is attentive for these alterations in patients complaining of diziness.

This pilot study still bears insufficient data for a statistical treatment able to judge the glucose intolerance test, and its values established by the American Association of Diabetes, which only considers time points zero and 120 minutes. The relevant findings, such as reactional hypoglycemia in intermediate time points between zero and 120 minutes, together with result agreements found in the specific literature, lead us to increase the number of patients studied with this pathology, so commonly found in our clinical practice.

## CONCLUSION

The data obtained in the assessment of the groups allows us to conclude that:

Diziness is a good indicator of glucose metabolism alterations. Glucose metabolism alteration is a good indicator of alterations in the vestibular test. Glucose metabolism studies based on glycemia levels is efficient and has results which are close to those observed in insulinemia curve tests. This study has to be broadened in order to make its findings relevant.
